# Preclinical Safety and Pharmacokinetics of Heat Stable Oxytocin in Sublingual Fast-Dissolving Tablet Formulation

**DOI:** 10.3390/pharmaceutics14050953

**Published:** 2022-04-28

**Authors:** Changcheng Zhu, Manjari Lal

**Affiliations:** Medical Devices and Health Technologies, PATH, Seattle, WA 98121, USA; czhu@path.org

**Keywords:** oxytocin, PPH, thermostable, sublingual, tablet, fast dissolving, rapid response, administration, needle-free

## Abstract

The work reported here focuses on an evaluation of a novel heat stable formulation of a uterotonic peptide drug oxytocin involving stability testing under elevated temperatures and toxicokinetic response generated by sublingual (SL) administration in rabbits. The formulation was thermotolerant, maintaining the potency of oxytocin in the form of a fast-dissolving tablet at the end of 2-year storage at 30 °C/65% relative humidity with less than 5% loss in oxytocin content based on analytical high performance liquid chromatography (HPLC). The toxicokinetic results in rabbits showed that the fast-dissolving tablet was safe without any reactogenicity or toxicity associated with SL administration or the excipients present in the formulation. The SL route elicited rapid absorption of oxytocin in plasma within 5 min of administration although lower than intramuscular (IM) administration. IM resulted in area under the curve (AUC) values approximately 5 times higher than SL oxytocin. However, due to the limitations encountered during SL administration in an anesthetized rabbit model, the relevance of heat stable oxytocin formulation that has the flexibility to be adapted in different formats may warrant a human clinical study to determine whether therapeutically relevant plasma levels for treating postpartum hemorrhage can be generated via alternate non-injectable routes of administration.

## 1. Introduction

An estimated 295,000 women die each year in childbirth [1], with long-term consequences for families and offspring. The majority of maternal deaths (94%) occur in low- and middle-income countries (LMICs), particularly in sub-Saharan Africa and South Asia. The leading direct cause of maternal death worldwide is hemorrhage, with the vast majority of these occurring during the postpartum period [2]. As per WHO, active management of the third stage of labor (AMTSL)—defined as intramuscular (IM) administration of 10 international units (IU) of oxytocin and controlled cord traction performed by a skilled birth attendant (SBA)—substantially reduces the risk of postpartum hemorrhage (PPH) [3].

Considering the severity of mortality and morbidity caused by PPH in low-resource settings (LRS), it is likely that a dosage form that is safe, easy to administer and provides a rapid and effective response could be more effective in saving lives in these settings. Oral is the most desirable route of administration due to accessibility and simplicity of the dosage form. However, for peptide drugs such as oxytocin, oral delivery displays low systemic availability and diminished efficacy due to the physiology of this route where high proteolytic degradation in the stomach and intestinal region degrades the peptide molecules in addition to the chemical degradation by the harsh pH environment encountered in the stomach region. Furthermore, the characteristics of injectable oxytocin, including its reliance on the cold chain, limit its reach in terms of effective coverage [4].

A heat-stable oxytocin in a fast-dissolving tablet (FDT) format for sublingual (under-the-tongue) delivery, offers easy administration of oxytocin with uncompromised quality (maintaining drug content), eliminating the need for injection and paving the way for community distribution in areas with limited health infrastructure, including potentially at-home births. In addition, heat-stable oxytocin as a SL FDT offers the favorable side effect profile of the WHO uterotonic drug of choice with the added advantage of being indicated potentially for both prevention and management of PPH.

The sublingual (SL) route offers a rapid onset of action [5] and direct delivery into the systemic circulation, bypassing oral gastrointestinal degradation and the first-pass metabolism in the liver [6]. This route is non-invasive and easily accessible, for administration by the caregiver, not requiring a trained health worker or a hospital setting as needed for parenteral (intramuscular (IM) or intravenous (IV)) administration of oxytocin. Furthermore, with the addition of excipients to impart a sweet taste to the product to improve palatability, this administration route anticipates better patient acceptability and compliance because the route does not involve conventional swallowing of the dosage form which may be difficult for woman undergoing PPH. Despite the obvious benefits, the sublingual route of administration has not been explored for delivery of oxytocin for the treatment of PPH.

Results from an initial feasibility study in minipigs reported previously by the authors show successful absorption of oxytocin via the SL route, along with improved thermostability of oxytocin in a fast-dissolving tablet form [7]. Considering the significance of the SL route, the objective of this study was to confirm the findings of the feasibility study by conducting a toxicokinetic study in an anesthetized rabbit model using an optimized formulation containing excipients safe for human use. Building upon the previous findings, we hypothesized that presentation of oxytocin as a heat-stable, sublingual fast-dissolving tablet (FDT) will be safe and non-reactogenic and will result in rapid absorption of oxytocin and generate both sufficient oxytocin levels in plasma and a long product shelf life under elevated temperature conditions as needed in areas with no or low cold storage facilities. Confirmation of these factors was considered to be a prerequisite for advancing a heat-stable formulation of oxytocin in a sublingual fast dissolving tablet into an exploratory clinical study.

## 2. Materials and Methods

### 2.1. Lyophilization Cycle for Production of Sublingual Oxytocin Fast-Dissolving Tablets

Heat-stable SL oxytocin tablets were produced using the lyophilization cycle used previously [7] as shown in Table 1. The formulation composition was similar to the previous formulation with one exception being replacement of carbomer with 0.25 wt% chitosan. To formulate the tablets, we used pharma grade Sucrose and Mannitol purchased from J. T. Baker (Phillipsburg, NJ, USA), Dextran-40 purchased from Pharmacosmos (Holbæk, Denmark) and pharma grade Chitosan purchased from Glentham Life Sciences (Corsham, UK). The size of the sublingual tablets were 8–10 mm diameter and 2–3 mm in thickness.

### 2.2. Sublingual Toxicokinetic Study Design

The toxicokinetic study in rabbits (Table 2) was conducted at a contract animal facility (Noble Life Sciences, Sykesville, MD, USA) under approval from Noble Life Sciences IACUC (approval code: NLS-589, date: 13 May 2020) and in compliance with the U.S. Food and Drug Administration Good Laboratory Practice Regulations for Nonclinical Laboratory Studies (21 CFR Part 58).

**Figure 1:**
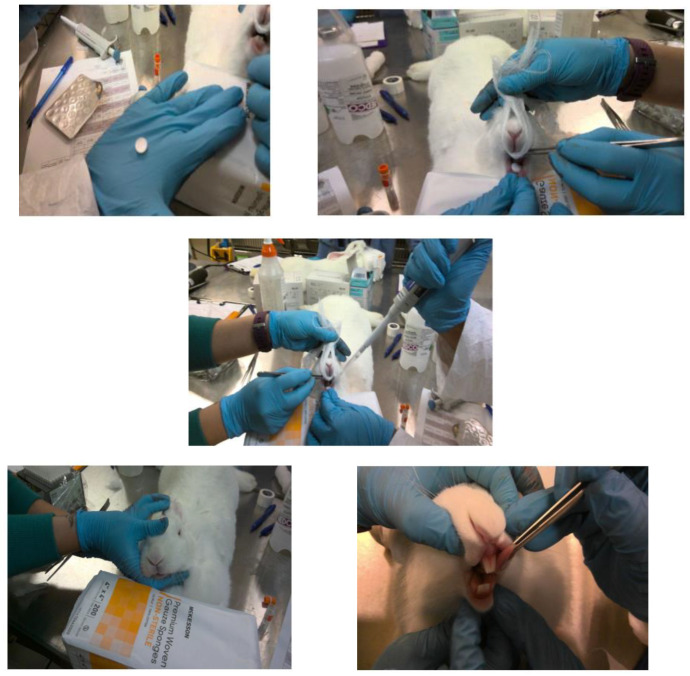
Placement of oxytocin fast-dissolving tablets in the sublingual cavity of anesthetized rabbits.

For SL placement of fast dissolving tablets, the rabbit was anesthetized and positioned on a flat table, with the lower jaw strongly supported in a horizontal level on the table. Using rounded tip stainless tweezers, the rabbit’s tongue was carefully raised, and the sublingual tablet was placed beneath the tongue (see Figure 1). Anesthesia was maintained for 3 h after drug administration to keep the tablet in sublingual position and prevent entering the gastrointestinal tract.

### 2.3. Administration Site Observations

A “Draize-like” scoring paradigm for injection site monitoring was used to grade the presence of erythema (and/or edema according to the reported method) [8]. In addition, any other change at the administration site (such as eschar, abscesses, necrosis, suppuration) was recorded when observed. Additionally, ulceration, blood, mucus, scars, and lesions were observed one hour before dosing, 3 h post-dose, and at euthanasia.

### 2.4. Blood Collection

Animals were not fasted prior to blood collection. Blood was collected at the time points of 0 min (pre-dose) and 5, 10, 15, 20, 30, 60, 120, and 180 min post-dose and at euthanasia only the first four animals for SL group. All animals were anesthetized pre-dose, prior to dosing. All animals were anesthetized with ketamine (35–50 mg/kg) and xylazine (3–5 mg/kg) via a subcutaneous or intramuscular (IM) route followed by maintenance anesthesia with additional half doses if needed or via inhaled isoflurane. At the selected time points, whole blood was collected aseptically from the auricular artery and dispensed into the appropriate collection tubes in any order: (1) K3 EDTA for hematology determinations; (2) 0.129 M, 3.8% sodium citrate for coagulation analyses (plasma); (3) serum separator tube for serum chemistry and C-reactive protein; and (4) aprotinin-spiked (K_2_EDTA) tubes for plasma oxytocin determinations. Tubes containing hematology specimens were placed at room temperature on a rocker for at least 30 min prior to shipment. Tubes containing whole blood for serum isolation were maintained for at least 30 min and no more than 4 h at room temperature before being processed to serum. Tubes containing aprotinin-spiked (K_2_EDTA) specimens for oxytocin determinations were maintained on wet ice or cold packs before use and after collection. They were also maintained on a rocker for at least 30 min prior to processing.

### 2.5. Plasma and Serum Processing

Blood samples for oxytocin determination were collected into the Aprotinin spiked (K_2_EDTA) vacutainer tubes and processed for collection of plasma. The samples were processed by centrifuging for approximately 15 min at approximately 3000 rpm and approximately 4 °C; they were aliquoted into cryovials without anticoagulant and were immediately placed on dry ice. At termination, blood was collected from the SL group for serum chemistry, C-reactive protein, hematology, and coagulation. Blood collected for coagulation analyses was collected into tubes containing 3.8% sodium citrate and was processed for plasma per the Noble protocol. All serum and plasma samples were processed aseptically.

### 2.6. Liquid Chromatography with Tandem Mass Spectrometry (LC-MS/MS) Analysis

Each plasma sample was aliquoted into two vials of an approximately equal volume. All the collected plasma was analyzed for oxytocin levels by the LC-MS/MS method.

The bioanalytical method for quantification of oxytocin in rabbit plasma (K_2_EDTA) was validated following the FDA guidelines [9] for a bioanalytical method validation at NorthEast BioAnalytical Laboratory, LLC (NEBA) (Hamden, CT, USA), via reverse phase liquid chromatography separation (Column: Zorbax SB C18 (Agilent, Santa Clara, CA, USA) 3.5 μm 150 × 4.6 mm, Security/Guard Cartridge Gemini C18 4 × 2.0 mm) and detection by tandem mass spectrometry (LC/MS/MS) using Sciex API-5000 Mass Spectrometer. Analysis of Oxytocin was performed using stable label internal standard, Oxytocin-d5. Oxytocin was received as an acetate salt and Oxytocin-d5 was received as Trifluoroacetate salt.

For quantitation, the LC/MS/MS data were acquired using Analyst^®^ v1.6 software (Sciex, Framingham, MA, USA). The calibration curves for Oxytocin were constructed as a non-linear function of the weighted (1/x^2^) linear regression of the theoretical concentration versus response (where response = ratio of analyte peak area to internal standard peak area) using the following equation:

*y* = *mx* + *b*

where:

*y* = peak area ratio of analyte/internal standard

*m* = slope of the corresponding standard curve

*x* = concentration of analyte (pg/mL)

*b* = intercept of the corresponding standard curve

Accuracy and precision were calculated using the following formulas

$$\begin{array}{c} \%\;\mathrm{Accuracy}=\;\frac{Calculated\;concentration}{Theoretical\;concentration}\;\times \;100\\ \%\;\mathrm{CV}=\;\frac{Standard\;deviation\;\left(SD\right)}{Mean\;measured\;concentration}\;\times \;100 \end{array}$$

Precision:

Accuracy was calculated using theoretical concentrations. Accuracy and precision were reported with one (1) decimal place. All concentration data are reported to two (2) decimal places. Excel^®^ was used for formatting data tables and computing summary statistics.

### 2.7. Pharmacokinetic Analysis

A sublingual FDT containing 400 IU oxytocin was administered in rabbits. A 10 IU dose of oxytocin in the form of IM injection was used as a control. The serum peak concentration (C_max_) and time-to-peak concentration (T_max_) were taken from the plasma concentration time profiles. Plasma concentration (μg) versus time (h) profiles were prepared and peak plasma concentration (C_max_) and time of its occurrence (t_max_) were read directly from the respective profiles. The area under the concentration time curve (AUC_0→t_) was calculated according to linear trapezoidal method using GraphPad Prism version 4.

### 2.8. Good Laboratory Practice Sublingual Reactogenicity/Toxicity Study

On Day 1, after the sample collection, all animals were euthanized and the first four animals in the SL group were subjected to a necropsy. Protocol-required tissues were processed, embedded in paraffin, sectioned, and stained with hemotoxylin and eosin (H&E) at Histo-Scientific Research Laboratories (HSRL) (Mount Jackson, VA, USA). The following tissues from the SL group were preserved in 10% neutral buffered formalin.

1. Appendix
2. Cecum
3. Colon
4. Duodenum
5. Esophagus
6. Ileum
7. Jejunum
8. Rectum
9. Sacculus rotundus
10. Sublingual tissue

## 3. Results

### 3.1. Characterization and Physical Stability of Sublingual FDTs

**Figure 2:**
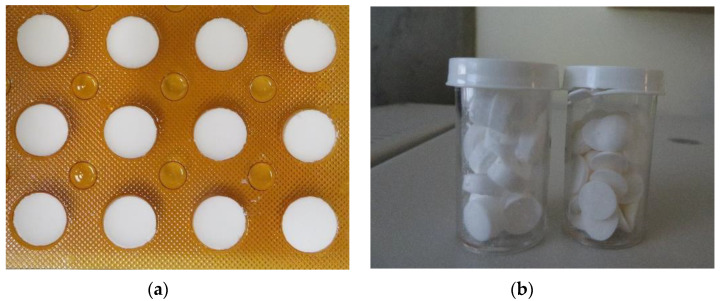
Sublingual oxytocin optimized formulation: (**a**) freeze-dried oxytocin tablets in blister sheet; (**b**) representative freeze-dried tablets in bottles.

**Figure 3:**
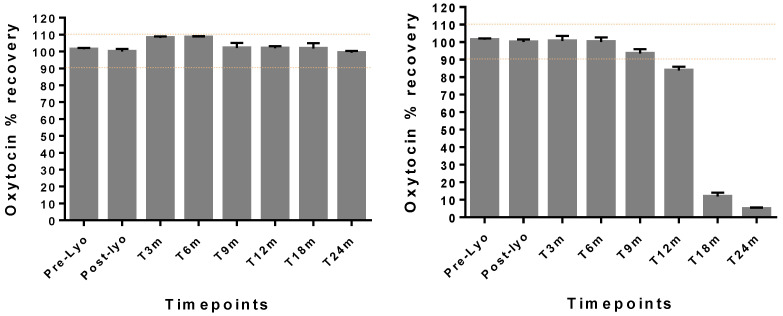
Stability data on heat-stable sublingual oxytocin tablets (*n* = 2 per time point) stored at (**left**) 30 °C/65% relative humidity (RH) and (**right**) 40 °C/75% relative humidity (RH) for 24 months. The Y-axis shows recovery in oxytocin content from tablets undergoing stability evaluation under different storage conditions. The yellow dotted lines indicate oxytocin content maintained within 90–110%.

Physical evaluation of the optimized oxytocin in sublingual FDT formulation containing chitosan showed that the tablets were robust with good handling properties for oral disintegration dosage forms based on friability (less than 3 wt%) and hardness (30N) testing using TH3 tablet hardness tester by Copley Scientific (Nottingham, UK) and FT2 friability tester from Sotax Corporation (Aesch, Switzerland), respectively (data not shown). Overall, the results demonstrate that the FDTs maintained their physico-chemical stability with a disintegration time under 30 s and remained stable, maintaining the oxytocin content within 90–110% for a period of 24 months under 30 °C/65% RH storage with minimal loss due to degradation based on HPLC assay (see Figure 2 and Figure 3). At 40 °C/75%RH, the tablets maintained the oxytocin content for up to 9 months, rapidly degrading after 12 months with a loss >90% at the end of 24 months. The moisture content in these FDTs was below 3 wt% for lead formulation (Oxy-004) after incubating at 30 °C/65%RH for 2 years. The tablets stored in sealed blister sheets did not show any change in color or appearance throughout the storage period.

### 3.2. Good Laboratory Practice (GLP) Toxicokinetic Study

**Figure 4:**
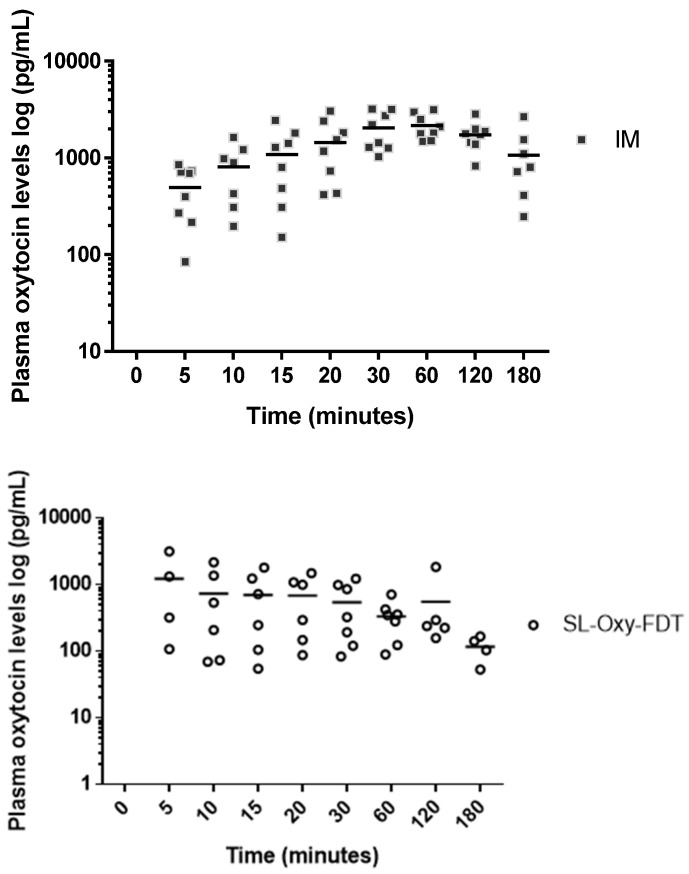
Individual plasma levels of oxytocin obtained via intramuscular (IM) and sublingual (SL) fast-dissolving tablet (FDT) route of administration in rabbits. Green line: Mean for SL group, Red line: Mean for IM group.

The mean plasma levels (measured by LC-MS/MS) obtained after SL or IM administration of oxytocin in a rabbit model are shown in Figure 4 and the pharmacokinetic parameters are presented in Table 3 below. As seen in Figure 4, a rapid increase in plasma oxytocin concentration was observed within 5 min for both SL- and IM-dosed groups. However, in the SL group, only 4/8 animals responded, while for the IM group, 8/8 animals responded, though the mean plasma oxytocin level was found to be higher for the SL group within the first 5 min of administration. The oxytocin levels in plasma continued to rise between 20 and 60 min after administration for both groups, whereas the overall plasma levels obtained were lower for the SL group than for those obtained with IM administration. Exposures following 10 IU doses of oxytocin in the IM control group resulted in low inter-subject variability compared to SL oxytocin, which showed high inter-subject variability and limited exposure. The total exposure of the body to oxytocin via the SL route was observed to be 1234 pg/h/mL compared to 4822 pg/h/mL in the control IM group (Table 4), suggesting lower drug exposure for the SL-dosed animals.

### 3.3. Good Laboratory Practice (GLP) Local Reactogenicity and Safety Study

**Figure 5:**
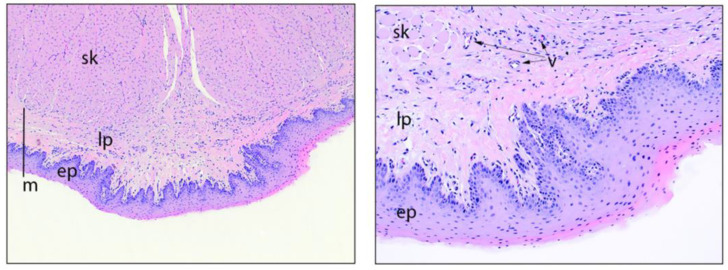
**Left**: tongue, ventral surface, 4x objective magnification. The ventral tongue near the midline appears microscopically normal after sublingual test article dosing. The dark bar denotes the two layers of the mucosa (m), which are the lamina propria (lp) and the epithelium (ep). sk = skeletal muscle fibers. **Right**: tongue, ventral surface, 10× objective magnification, at a higher magnification view of the mucosa, the lamina propria (lp) consists of wavy collagen fibers with occasional embedded arterioles and capillaries (v = vessels) but no notable leukocytic infiltrate. The epithelium (ep) is a stratified squamous and non-keratinized. sk = skeletal muscle fibers.

Animals that underwent necropsy were within the normal clinical limits of body weight, body temperature, blood pressure, heart rate and food consumption, at the time of gross necropsy. Overall, the tablet administration site (sublingual tissue on the tongue) and alimentary tract tissues appeared to be normal and showed no gross observational changes. In all cases, the tongue ventral surfaces appeared normal after administration of the oxytocin in sublingual FDT formulation (Figure 5). There were no unscheduled deaths among the animals submitted for histopathological evaluation. There were no macroscopic or microscopic observations reported at necropsy in the sublingual tissue or alimentary tract of rabbits terminated 24 h after SL administration of oxytocin tablet.

### 3.4. Sublingual Administration Site Observations

There were no findings in erythema or edema scores at either administration site during the study. All animals had 0 scores (no erythema or edema) at the injection site at various time points throughout the study.

## 4. Discussion

Our goal was to validate the findings from the previous feasibility study [7] by using a preclinical rabbit model and a formulation that was optimized for potential use in humans by using excipients which are Generally Recognized As Safe (GRAS), with low or minimal toxicity or reactogenicity. We also assessed the optimized formulation for thermostability of oxytocin and pharmacokinetic behavior when administered via the sublingual route. In the previous feasibility study, we used carbomer as the mucoadhesive agent and sodium taurocholate as the permeability enhancer for oxytocin (by potentially altering the lipid bilayer fluidity, modulating the tight junction permeability to help oxytocin cross the epithelium). In this study, we attempted two approaches to optimize the previously reported formulation: (1) was replaced sodium taurocholate with pharmaceutical grade sodium cholate [10,11], as a permeation enhancer; and (2) we replaced carbomer and sodium taurocholate with chitosan [12,13,14], which has both mucoadhesive and permeation-enhancing properties. During formulation preparation, we observed that carbomer is light and fluffy and tends to float on water, requiring a good vortex to become thoroughly wet. It requires great accuracy during mixing in order to avoid irregular dispersion of the powder, leading to the formation of lumps and heterogeneous samples. In addition, due to the acrylic acid backbone, when hydrated, this polymer forms acidic solutions that become increasingly viscous and gel-like as the pH is adjusted to above 4 (as needed for oxytocin). This poses a difficulty, especially during formulation preparation and scale-up. The carbomer formulation also generated residue in the blister sheet upon removal of the tablets. Due to these undesirable formulation attributes, the formulation containing carbomer was not selected as the lead formulation in this study.

Chitosan, a natural cationic polysaccharide, has received a great deal of attention in the past few years for its mucoadhesive character [15], permeation enhancing properties, and controlled release of drugs. It is recognized by the Food and Drug Administration as GRAS [16,17], as listed in the Code of Federal Regulations (CFR 21). Since the formulation was aimed at developing a dosage form, that was robust, heat stable, rapid dissolving, and which could provide quicker onset of action, the developed sublingual formulation met all the criteria. In addition, there were no test article-related adverse changes in mortality, clinical observations, injection site scores, body weights, food consumption, fecal output, heart rate, blood pressure, or body temperature. The necropsy at 24-h post dosing and histopathology in sublingual and alimentary tract tissues were normal. This is important as during sublingual administration, there is an involuntary swallowing via the gastro-intestinal route. However as noted, the formulation showed no apparent test article-related microscopic findings in the sublingual tissue or the alimentary tract of rabbits who underwent necropsy. There were no abnormal findings in the clinical pathology data that could be attributed to the sublingual administration of oxytocin containing FDT formulation.

It is important to highlight that in the pharmacokinetic study, with SL administration, the absorption of oxytocin was rapid, with a mean absorption time within about 5 min of administration. Rapid absorption is a great advantage for the SL route because it is legitimate to assume that, if C_max_ is obtained quickly, the biological effect of uterus contraction as needed to reduce bleeding (during PPH) would also be obtained quickly. As per the World Health Organization guidelines, oxytocin is administered either via IM or IV, and each route has potential advantages. The IV administration has a clinical advantage, as it leads to a faster response and a higher peak in plasma oxytocin levels; however, IM injection confers practical advantages, requiring fewer skills and less equipment to administer, making it a more serviceable option in a wider array of settings. Considering the rich vasculature in the SL region, where the blood vessels under the tongue drain into the jugular vein, rapid absorption of oxytocin into the blood circulatory system via the SL route is expected and in principle would mimic IV injection by introducing a drug directly into the systemic circulation.

The results from the pharmacokinetic study in rabbits showed that, although the SL route appeared to be associated with rapid absorption of oxytocin in plasma, the overall bioavailability was significantly lower with a higher variability among animals within the SL-dosed group as compared to the IM control group. This means that, despite the high sublingual dose of 400 IU, only a fraction of oxytocin was being absorbed via the sublingual route. The maximum concentration of oxytocin obtained via the SL route was 1164 pg/mL; for the control group (IM administration to anesthetized rabbits), the maximum oxytocin concentration was 2662 pg/mL. Specifically, for SL formulations, the plasma levels (C_max_) pg/mL obtained were about 30% to 45% of the oxytocin levels obtained via the IM route (control). In a study conducted in 2004 by Carvalho et al. [18,19], the authors stated the minimum effective initial dose of oxytocin to minimize blood loss and to prevent PPH in elective caesarean deliveries to be 0.35 IU, which was far less than the previous conventional dosages ranging from 5 to 10 IU, suggesting the necessity of further research into the administration of oxytonics in a rational and judicious manner to minimize the side effects while maintaining efficacy. Similarly, another clinical study suggested that excessive doses of oxytocin to achieve an adequate uterine tone needs re-evaluation and observed that the adequate uterine tone can be achieved with small bolus doses like 0.5–3 IU of oxytocin. In view of these observations, it is possible that even the low plasma oxytocin levels obtained via the SL route in this study may still be therapeutically relevant in treating PPH; however, this can only be confirmed by testing in a relevant PPH preclinical model.

In our study, it is difficult to ascertain whether the lower oxytocin levels were due to the effect of anesthesia, which may have impacted available saliva, resulting in improper or incomplete absorption of the dose, or if they were due to the structural constraints imposed by oxytocin, limiting the absorption via passive diffusion across the sublingual mucosa. The effect of anesthesia has been previously noted by the authors where by administering the sublingual tablet in unanesthetized animals, the plasma levels of oxytocin obtained were higher, although they were inconsistent due to chewing (oral swallowing) of the tablets by the awake animals (PATH internal data), indicating the importance of a correct administration approach with the SL route of delivery.

Lastly, the optimized formulation containing chitosan was robust, with good handling properties. This formulationmaintained oxytocin drug content in the range of 90–110% drug content as per the USP monograph acceptable potency criteria for oxytocin [20,21] under ambient conditions (30 °C/65%RH) with a high humidity for up to 2 years. The WHO guidelines on stability testing of pharmaceutical products require that manufacturers of products to be stored outside the refrigerator and intended for sale in hot climatic regions (i.e., climatic zones III and IV) should demonstrate adequate stability at 30 °C (not 25 °C) over the intended shelf-life of the product. In this study, even under extreme storage temperatures of 40 °C, oxytocin was stable for at least 9 months, suggesting the protection offered by the stabilizing excipients present in the optimized formulation [22].

The present study, as is obvious, has some limitations as correlated with the pharmacokinetic study. First, the rabbits had to be anesthetized for SL administration and therefore, there was limited saliva volume available for tablet disintegration, requiring external administration of fluid 0.25 mL to assist with tablet disintegration. Although the smaller volume had a lower chance of inducing swallowing and/or loss of oxytocin, in an anesthetized animal, it was difficult to “hold” the fluid in place. This may have contributed to involuntary loss in dose, thus contributing to a lower available dose and lower plasma levels of oxytocin, despite the high SL dose in the formulation.

## 5. Conclusions

In conclusion, the authors have successfully developed a thermo-tolerant form of oxytocin in the form of a fast dissolving tablet for sublingual administration. The sublingual tablet was shown to be safe and demonstrated no reactogenicity or local toxicity in sublingual tissues or other organs in the gastro-intestinal region. The SL route appeared easy to administer and was well tolerated by the animals and characterized by minimal cardiorespiratory and other physiological effects. Due to the lower plasma oxytocin levels, it is unlikely that this administration route will elicit a relevant therapeutic effect as needed for the treatment of PPH. However, the absorption of oxytocin administered via the SL route was rapid, within 5 min of administration. This attribute needs further exploration, which could include an investigational clinical study to assess the potential of sublingual oxytocin for the treatment of mental health conditions such as postpartum depression or generalized anxiety and depression [23].

## Figures and Tables

**Table 1:** Freeze-drying cycle for production of heat-stable sublingual oxytocin.

Step	Temperature	Ramp (min)	Hold (min)	Vacuum
Freeze	−45 °C	0	180	NA
Primary drying	−40 °C	0	600	100 MT
	−10 °C	360	600	100 MT
Secondary drying	25 °C	240	600	100 MT
	30 °C	26	300	100 MT
Storage	4 °C	NA	NA	100 MT

**Table 2:** Rabbit toxicokinetic study design for evaluation of heat-stable sublingual oxytocin.

Group Designation	N	Dose Volume (Day 0)	Route	Time Points for Blood Collection (min)	Terminal Blood Collection	Necropsy
Oxytocin control (10 IU)	8	0.2 mL	IM	0, 5, 10, 15, 20, 30, 60, 120, 180	Not performed	Not performed
Sublingual oxytocin (400 IU)	8	One tablet + 0.25 mL water	SL	0, 5, 10, 15, 20, 30, 60, 120, 180	First four animals only	First four animals only

**Table 3:** LC-MS/MS method validation summary table.

Analyte	Oxytocin	
Matrix	Rabbit plasma (K_2_EDTA)	
Sample volume	400 μL	
Sample storage	−70 °C	
Extraction type	Protein precipitation	
Internal standard	Oxytocin IS	
Standard curve	50.25–10,001.38 pg/mL	
Quality control	QC_LLOQ	50.25 pg/mL
	QC_Low	149.92 pg/mL
	QC_Mid	799.57 pg/mL
	QC_High	7995.68 pg/mL
Regression weighting	Quadratic, weighted 1/x^2^	

**Table 4:** Comparison of mean oxytocin pharmacokinetic parameters for intramuscular versus sublingual formulation.

Group Dose Route/Formulation	Intramuscular (Control); *n* = 8	Sublingual Oxytocin Fast-Dissolving Tablet; *n* = 8
Dose (IU)	10	400
T_max_ (h)	1.13 ± 0.916	0.931 ± 0.642
C_max_ (pg/mL)	2662 ± 567	1164 ± 1179
AUC_last_ (pg·h/mL)	4822 ± 728	1234 ± 1001
T_last_ (h)	2.88 ± 0.354	2.50 ± 0.837
T_1/2_ (h)	1.02 ± 0.336	0.906 ± 0.333

## Data Availability

Data and materials are available by the authors by request.
